# Effects of a 12-Year Nitrogen Addition Experiment on Protist Communities in a Boreal Forest, Heilongjiang Province, China

**DOI:** 10.3390/ani16111734

**Published:** 2026-06-04

**Authors:** Gang Fu, Guancheng Liu, Ligong Wang, Yuguo Gao, Zhicheng Yao, Yajuan Xing, Qinggui Wang

**Affiliations:** 1 School of Life Science, Qufu Normal University, Qufu 273165, China; 18865471727@163.com (G.F.); lxtxlgc123@126.com (G.L.); 2 Shandong Key Laboratory of Wetland Ecology and Biodiversity Conservation in the Lower Yellow River, Qufu Normal University, Qufu 273165, China; 3 Daxing’anling Institute of Agriculture and Forestry, Jiagedaqi 165000, China; 13845747950@163.com; 4 Shandong Weishan Lake Wetland Ecosystem National Positioning Observation and Research Station, Jining 272000, China; gyg2000@yeah.net; 5 Zibo Forestry Protection and Development Center, Zibo 255000, China; yaozhicheng1985@163.com

**Keywords:** nitrogen addition, soil protists, functional groups, boreal forest, gene sequencing

## Abstract

Escalating nitrogen (N) deposition has profound effects on terrestrial ecosystems; however, the mechanisms underlying soil protist community responses in cold-temperate regions remain poorly understood. This study investigated the effects of long-term N addition on soil protist communities at Nanwenghe Reserve in the Greater Khingan Mountains, where a 12-year continuous N addition experiment was initiated in 2011 and has been maintained annually thereafter. Four N addition levels were applied: control (CK, 0), low N (LN, 25), medium N (MN, 50), and high N (HN, 75 kg N ha^−1^ yr^−1^). During the 2023 growing season, soil protist communities were characterized using 18S rRNA gene sequencing. Our results demonstrated that N addition significantly altered the protist community structure, with significant interactive effects between N addition and season. Phagotrophic and phototrophic protists exhibited significantly stronger responses to N addition than parasitic protists. Phototrophic communities showed the strongest compositional differentiation (NMDS, *p* = 0.002), whereas parasitic protists exhibited no significant response to N addition. PLS-PM analysis indicated that soil nutrient factors primarily exerted indirect effects on protist communities by altering microbial diversity and plant characteristics, whereas biotic factors, particularly fungal and plant diversity, showed stronger direct regulatory effects on protist community dynamics. These findings reveal how N addition influences soil protist communities through the soil–plant–microbial pathway, providing a scientific basis for assessing soil microbial food web stability in high-latitude forests.

## Simple Summary

Long-term nitrogen (N) deposition affects cold-temperate forest ecosystems; however, the mechanisms underlying soil protist responses remain unclear. Based on a N addition platform established in 2011 in the Greater Khingan Mountains (0, 25, 50, 75 kg N ha^−1^ yr^−1^), we analyzed protist communities using 18S rRNA gene sequencing. Results showed that N addition significantly altered community structure, with effects varying significantly by season. Phagotrophic and phototrophic protists were sensitive to N addition, whereas parasitic protists exhibited no significant responses. Soil nutrients, fungal diversity, and plant diversity collectively influenced community dynamics. This study reveals how N addition regulates protist communities via the soil–plant–microbial pathway.

## 1. Introduction

Nitrogen (N) is a key nutrient limiting primary productivity in terrestrial ecosystems, playing a central role in driving soil biogeochemical cycles and maintaining ecosystem stability [1]. Since the Industrial Revolution, fossil fuel combustion and intensive agricultural activities have greatly increased atmospheric N deposition worldwide. Although global N deposition peaked around 2015, current deposition rates still remain approximately two to three times higher than pre-industrial atmospheric N deposition levels [2]. Long-term N deposition can alter soil physicochemical properties, particularly soil pH, soil organic carbon content, and inorganic N availability; alleviate N limitation; and induce trophic cascades, thereby reshaping soil biological communities and influencing ecosystem carbon and nutrient cycling [3].

Soil protists are core components of the soil microbial food web. As major predators of bacteria and fungi, they play important roles in regulating microbial community structure, accelerating nutrient mineralization, and maintaining soil ecosystem functioning [4]. Based on nutritional strategies, protists can be classified into phagotrophic, parasitic, and phototrophic functional groups, which exhibit distinct response patterns to environmental changes [5]. Specifically, phagotrophic protists promote nutrient turnover through microbial predation; parasitic protists regulate host population dynamics, and phototrophic protists possess both autotrophic and heterotrophic metabolic features that confer strong adaptability to soil nutrient changes [6,7,8]. Therefore, elucidating the differential responses of protist communities and their functional groups to N addition is crucial for revealing the mechanisms maintaining soil microbial food web stability and understanding how N deposition regulates soil ecosystem functioning.

Existing studies have shown that the effects of N addition on soil protist communities vary among ecosystems. These effects involve both direct influences on soil properties and indirect influences through food-web interactions [6,9]. Direct effects occur when N input alters soil pH, increases ammonium toxicity, or alleviates nutrient limitation, thereby exerting stimulatory or inhibitory effects on protists [10]. Indirect effects occur through trophic cascades, whereby changes in microbial prey availability and food quality subsequently alter protist community composition and functional dynamics [11,12]. In temperate grasslands and subtropical montane rainforests, N addition typically reduces protist diversity and simplifies community structure, primarily due to the negative impacts of soil acidification and carbon-to-nitrogen imbalance on the microbial food web [13,14]. In subtropical forests with N limitation, moderate N addition can enhance protist diversity indirectly by relieving nutrient constraints and promoting microbial growth, whereas excessive N input often causes nutrient imbalance and significant inhibitory effects on protist communities [15,16]. Compared with mid- and low-latitude ecosystems, the cold-temperate *Larix gmelinii* forests of the Greater Khingan Mountains exhibit distinct biogeochemical characteristics: low temperatures, slow organic matter decomposition rates, and relatively conservative carbon and N cycling, all of which promote substantial organic matter accumulation in forest soils [2]. These unique features may strongly influence soil microbial food webs and alter protist responses to N deposition. Consequently, the divergent responses observed across ecosystems preclude direct extrapolation of findings from mid- and low-latitude regions to high-latitude cold-temperate forests, underscoring the need for targeted research in the Greater Khingan Mountains.

Building upon this background, the present study was conducted at a long-term N addition experimental site established in 2011 in the Greater Khingan Mountains. Using high-throughput sequencing of 18S rRNA, 16S rRNA, and ITS genes, we investigated the effects of long-term N addition on soil protist communities and their functional groups in a cold-temperate boreal forest ecosystem, and further explored how soil nutrients, microbial diversity, and plant characteristics jointly regulate protist community dynamics. The core scientific questions addressed herein are as follows: (1) How does long-term N addition alter the diversity and community structure of soil protists in boreal cold-temperate forests? (2) Do phagotrophic, parasitic, and phototrophic protist functional groups show divergent responses to N addition and seasonal variation? (3) What key abiotic and biotic factors drive the dynamics of protist communities under N enrichment? Accordingly, three hypotheses were proposed: (1) N addition significantly affects soil protist community structure; (2) different functional groups exhibit distinct responses to N addition; and (3) protist community structure is jointly influenced by abiotic soil factors (soil nutrients) and biotic factors (microbial and plant diversity).

## 2. Materials and Methods

### 2.1. Study Site

This study was conducted at a long-term nitrogen (N) addition experimental site in Nanwenghe National Nature Reserve, the Greater Khingan Mountains, Heilongjiang Province, China (51°05′–51°39′ N, 125°07′–125°50′ E). The site lies on the southern slope of the Yilehuli Mountains, with an elevation of 500–800 m. It features a cold-temperate continental monsoon climate, with a mean annual temperature of −2.7 °C, a mean annual precipitation of 500 mm (80% concentrated in July–August), and a frost-free period of 90–100 days. The zonal vegetation consists of pure stands of *Larix gmelinii*, accompanied by Betula platyphylla. The soil type is sandy loam and gravelly sand.

### 2.2. Experimental Design and Sample Collection

**Figure 1:**
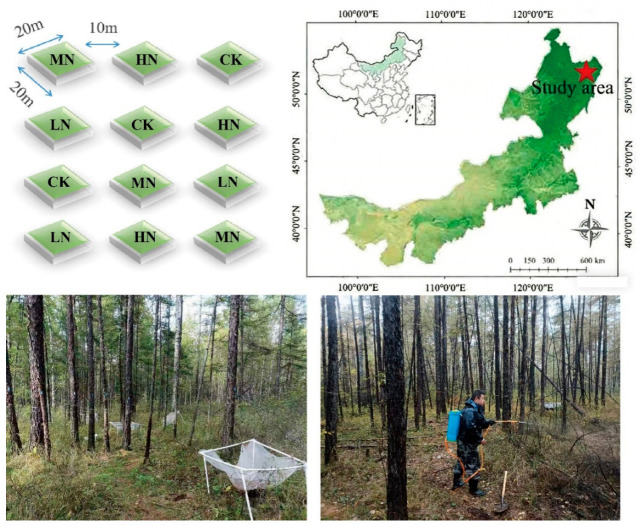
Distribution of N addition plots and experimental design in boreal forest ecosystems of the Greater Khingan Mountains in Northeast China. CK, control; LN, low nitrogen; MN, medium nitrogen; HN, high nitrogen.

The N addition experiment was initiated in 2011 using a randomized block design. Four N addition levels were established: control (CK, 0), low N (LN, 25 kg N ha^−1^ yr^−1^), medium N (MN, 50 kg N ha^−1^ yr^−1^), and high N (HN, 75 kg N ha^−1^ yr^−1^), which were set according to the background atmospheric N deposition rate in boreal forests and future deposition increase scenarios, corresponding to approximately 1×, 2×, and 3× the ambient N deposition rate in boreal forests, respectively. Each treatment had three replicates, yielding 12 plots in total, with 8–10 m buffer zones between plots (Figure 1). From 2011 to 2023, N was applied monthly during the growing season (May–September) each year. Litter layers were retained without removal during N application and soil sampling. NH_4_NO_3_ was dissolved in 32 L of deionized water for each plot according to the designated N level and sprayed evenly onto the plot surface using a hand-held sprayer. The control plots received an equivalent volume of deionized water to eliminate potential confounding effects of water addition (Figure 1). All plots were located within the same forest stand and shared similar topographic and vegetation conditions, thereby minimizing environmental heterogeneity unrelated to N addition. Soil water content was measured during each sampling period and included in subsequent analyses. Soil temperature and litter inputs were not experimentally manipulated, but all plots experienced the same natural climatic conditions and forest management regime throughout the study period.

Soil samples were collected in May, July, and September 2023, corresponding to the early, middle, and late growing stages of local plants. The understory vegetation is dominated by tree seedlings (*Quercus mongolica*), shrubs (*Rhododendron dauricum*, *Spiraea salicifolia*, *Rubus saxatilis*, *Rosa rugosa*, *Lespedeza bicolor*), herbs (*Deyeuxia langsdorffii*), and fern (*Pteridium aquilinum*). After removing surface litter, topsoil (0–10 cm) was collected using a five-point composite sampling method within each plot. The five subsamples were mixed to form one composite sample per plot, yielding 36 samples in total (4 treatments × 3 replicates × 3 months). Each sample was divided into two portions after removing visible roots and debris: one portion was stored at 4 °C for physicochemical analysis, and the other was frozen at –80 °C for DNA extraction and molecular sequencing. Concurrently, three 1 m × 1 m quadrats were established in each plot to survey plant species composition.

### 2.3. Physicochemical Analyses and Sequencing

Detailed protocols for soil physicochemical analyses are provided in Supplementary Materials Tables S1–S3.

Total genomic DNA was extracted from soil samples using the E.Z.N.A.^®^ Soil DNA Kit (Omega Bio-tek, Norcross, GA, USA). DNA quality and integrity were detected by a NanoDrop 2000 spectrophotometer (Thermo Fisher Scientific, Wilmington, DE, USA) and 1% agarose gel electrophoresis. The specific primer pair targeting the 18S rRNA V4 region was TAReuk454FWD1 (5′-CCAGCASCYGCGGTAATTCC-3′) and TAReukREV3 (5′-ACTTTCGTTCTTGATYRA-3′). Barcoded primers were used to amplify the bacterial 16S rRNA (V3–V4), fungal ITS (ITS1), and protist 18S rRNA (V4) regions. PCR amplification was performed in triplicate with Phusion High-Fidelity PCR Master Mix (New England Biolabs, Ipswich, MA, USA) under optimized annealing temperatures. The PCR products were pooled, purified, and quantified uniformly. Equimolar amplicons were sequenced on the Illumina MiSeq platform with 2 × 300 bp paired-end sequencing. Raw reads were quality-trimmed and merged, and chimeras were eliminated. Bacterial and fungal sequences were clustered into ASVs, whereas protist sequences were grouped into OTUs at a 97% similarity threshold using the UPARSE algorithm [17,18,19,20]. Taxonomic annotation was performed against the PR2 5.0 database. During bioinformatic filtering, OTUs assigned to Metazoa, Fungi, and Streptophyta at the kingdom level were excluded to remove non-protist sequences. Low-quality reads, chimeric sequences, and singleton OTUs were also removed during sequence processing and quality control. After filtering, a total of 738 protist OTUs were retained for downstream analyses. Referring to trophic information from the PR2 database and previous research on soil protist ecology [5,7,8], we categorized protist OTUs into phagotrophic, phototrophic and parasitic functional groups. The grouping was defined by the dominant nutritional lifestyles and ecological features of each taxonomic lineage.

### 2.4. Statistical Analyses

Generalized linear mixed-effects models (GLMMs) and linear mixed-effects models (LMMs) were used to evaluate the effects of N addition, season, and their interaction on soil microbial α-diversity. Species richness and abundance were analyzed using negative binomial GLMMs, while Shannon diversity was analyzed using Gaussian LMMs after log_10_ transformation when appropriate. Blocks and plots were included as nested random intercepts (1|Block/Plot) to account for the experimental design structure. Significance of fixed effects was assessed using Type II F-tests. Statistical analyses were performed in R v4.4.0 using the lme4, lmerTest, and car packages [21,22,23,24].

Protist α-diversity metrics were calculated using the vegan package (v2.6-6). Community structure was visualized using NMDS (Bray–Curtis), with stress values reported. PERMANOVA (adonis2, 999 permutations) was used to test compositional differences, preceded by PERMDISP to verify homogeneity of multivariate dispersions. Spearman correlation was used for bivariate relationships. PCA (prcomp, standardized) was applied to soil physicochemical indicators, and PC1 and PC2 (collectively explaining >65% variance) were extracted as composite soil nutrient variables [25,26,27].

Partial least squares path modeling (PLS-PM) was used to quantify direct and indirect pathways [28,29]. N addition and season were dummy-coded as categorical exogenous variables. Mediators included soil nutrient PCA axes, bacterial/fungal diversity, and plant characteristics; endogenous variables included protist α-diversity and relative abundance. Model quality was evaluated using GoF, SRMR, and R^2^. Path significance was assessed by bootstrapping (1000 resamples), and model robustness was further evaluated using cross-validation procedures to test predictive stability. All analyses were performed in R 4.4.0, except correlations in Origin 2023.

## 3. Results

### 3.1. Effects of Nitrogen Addition on Soil Physicochemical Properties and Microbial Communities

Nitrogen (N) addition and season significantly altered soil physicochemical properties (Supplementary Materials files: Tables S1 and S2). Both soil organic carbon (SOC) and plant taxonomic richness were stimulated under LN treatment but inhibited under HN treatment. Total phosphorus (TP) and microbial biomass carbon (MBC) decreased with increasing N addition levels, with MBC in HN treatment reduced to only 50% of that in CK treatment. Soil water content (SWC), pH, total nitrogen (TN), and total carbon (TC) all declined significantly with N addition. In contrast, dissolved organic carbon (DOC) was primarily influenced by season, increasing by 63.2–94.7% in July compared to May.

N addition significantly affected soil microbial α-diversity (Supplementary Materials file, Table S3). Both bacterial and fungal diversity increased under LN and MN treatments but decreased under HN treatment, with fungal diversity showing the most pronounced enhancement under MN treatment (Shannon index and richness increased by 39.9% and 47.8% relative to CK, respectively). Mixed-effects model results showed that N addition, season, and their interaction differentially affected microbial α-diversity across bacterial and fungal communities (Table 1).

### 3.2. Effects of Nitrogen Addition on Protist Communities and Functional Groups

**Figure 2:**
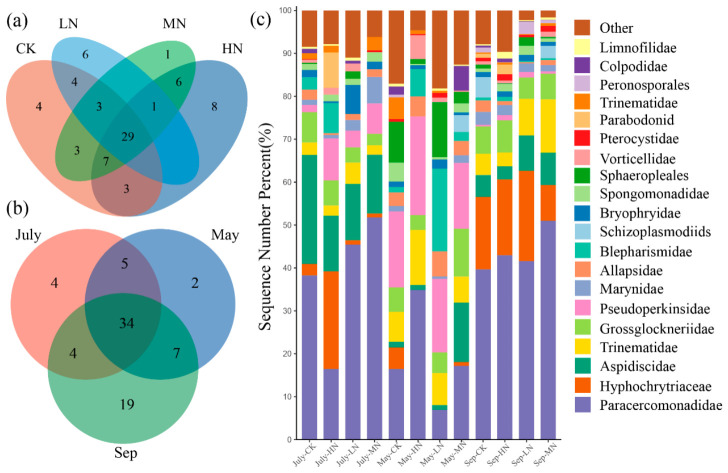
Venn diagrams showing the shared and unique protist families across nitrogen addition treatments (**a**) and sampling seasons (**b**), and relative abundance percentage bar charts (**c**) of protists at the family level. CK, control; LN, low nitrogen; MN, medium nitrogen; HN, high nitrogen. May, May; Jul, July; Sep, September.

Nitrogen (N) addition and season significantly influenced protist community composition. At the family level, shared and unique families were detected across all treatments, with MN treatment harboring the fewest unique families. Seasonally, the greatest number of shared families occurred between May and September, while the proportion of unique families increased markedly in September (Figure 2a,b). Phagotrophic protists dominated the community structure, with Paracercomonadidae and Hyphochytriaceae generally accounting for over 30% of relative abundance; Paracercomonadidae peaked at approximately 52% relative abundance in MN treatment in September. Parasitic groups (represented mainly by Pseudoperkinsidae) showed relatively limited responses to N addition. Among the taxa assigned to the parasitic functional group, Pseudoperkinsidae exhibited fluctuations in relative abundance among treatments in July, whereas no consistent response pattern was observed across seasons. Phototrophic protists (Sphaeropleales and Trinematidae) exhibited pronounced seasonal patterns, with overall higher abundance in September. In MN treatment, significant increases in Grossglockneriidae and Trinematidae elevated the proportional representation of this functional group (Figure 2c).

**Figure 3:**
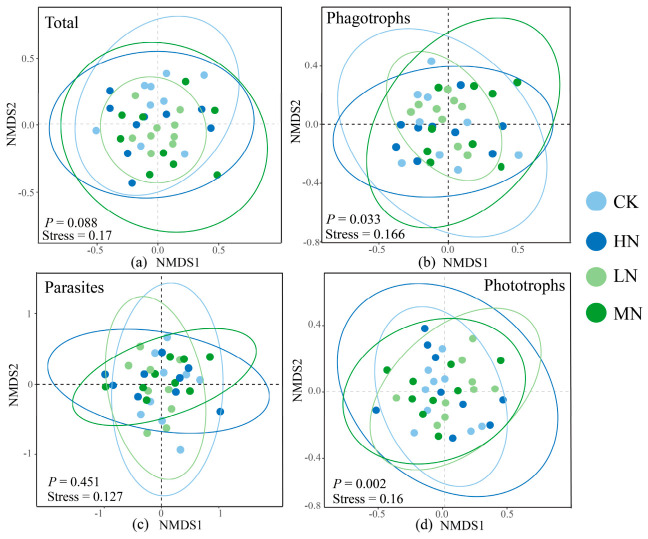
NMDS ordination of total (**a**), phagotrophic (**b**), parasitic (**c**), and phototrophic (**d**) protist communities under nitrogen addition. CK, control; LN, low nitrogen; MN, medium nitrogen; HN, high nitrogen.

NMDS analysis revealed that N addition altered the community structure of total protists and functional groups (Figure 3). To further evaluate differences in community composition among treatments, PERMANOVA analysis was conducted separately for each sampling month. The results demonstrated that N addition significantly affected protist community composition in May (R^2^ = 0.286, *p* = 0.021), July (R^2^ = 0.312, *p* = 0.015), and September (R^2^ = 0.295, *p* = 0.018), indicating significant treatment effects on protist community structure across sampling seasons. Total protist communities showed marginally significant differentiation (*p* = 0.088, α = 0.1). Phagotrophic communities exhibited higher differentiation significance than the total community, while phototrophic communities showed the strongest response (*p* = 0.002). In contrast, parasitic communities displayed no significant differentiation (*p* > 0.05).

**Figure 4:**
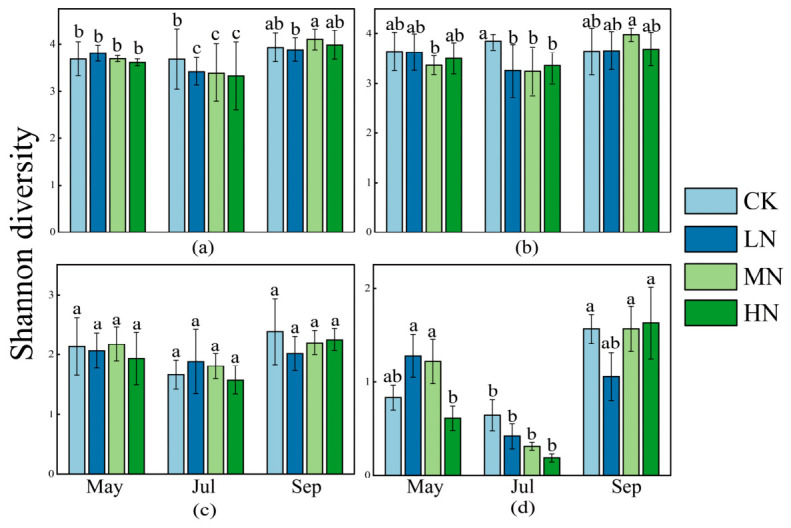
Shannon diversity of total (**a**), phagotrophic (**b**), parasitic (**c**), and phototrophic (**d**) protists under nitrogen addition and seasonal variation. Different lowercase letters indicate significant differences among N addition treatments within each season (*p* < 0.05, Tukey’s HSD test following simple effects analysis). CK, control; LN, low nitrogen; MN, medium nitrogen; HN, high nitrogen. May, May; Jul, July; Sep, September.

**Figure 5:**
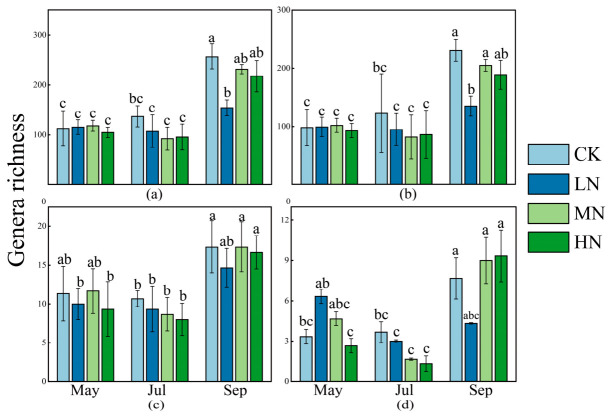
Richness index of total (**a**), phagotrophic (**b**), parasitic (**c**), and phototrophic (**d**) protists under nitrogen addition and seasonal variation. Different lowercase letters indicate significant differences among N addition treatments within each season (*p* < 0.05, Tukey’s HSD test following simple effects analysis). CK, control; LN, low nitrogen; MN, medium nitrogen; HN, high nitrogen. May, May; Jul, July; Sep, September.

N addition significantly affected α-diversity of total protists and all functional groups (Figure 4 and Figure 5; Table 2). Mixed-effects models indicated that N addition significantly influenced Shannon diversity and richness of total protists (*p* < 0.001), with their interaction significantly affecting richness (*p* < 0.001). Phagotrophic and phototrophic diversity were significantly impacted by N addition; most indices were also regulated by season, though the interaction was not significant. Parasitic richness was significantly affected by season only (*p* < 0.001). Shannon diversity of total protists and phagotrophs followed similar patterns, peaking in CK treatment in July, with MN and HN treatments significantly higher than CK and LN in September. Phototrophic diversity showed the most pronounced seasonal variation: significantly higher in LN treatment than CK and HN treatments in May, significantly reduced across all treatments in July, and significantly recovered in MN and HN treatments in September. Parasitic diversity did not differ significantly among N treatments.

### 3.3. Relationships Between Soil Protist Communities, Soil Physico-Chemical Properties and Microorganisms Under N Addition

Spearman correlation analysis revealed that diversity of total protist communities and each functional group was significantly correlated with soil physicochemical factors and microbial diversity (Figure S1). Specifically, Shannon diversity and richness of total protists were significantly negatively correlated with SOC, DOC, and pH, but significantly positively correlated with plant diversity and fungal diversity. Fungal diversity exerted stronger positive effects on most protist groups, whereas bacterial diversity was significantly correlated only with phototrophic protists.

**Figure 6:**
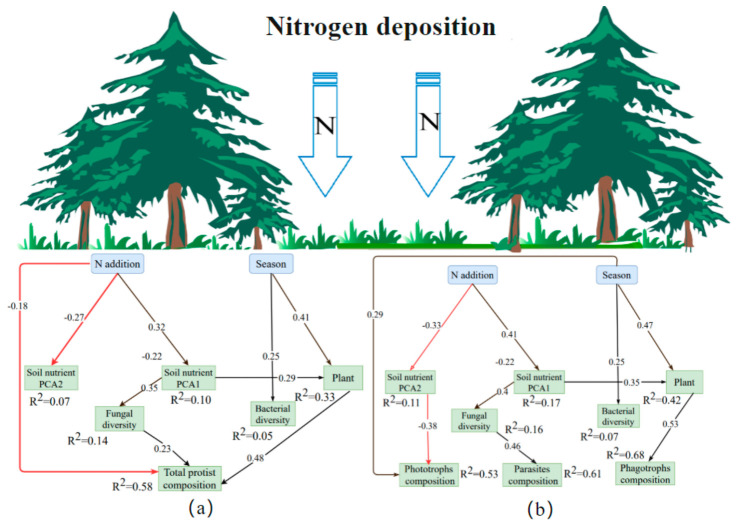
PLS-PM demonstrated the direct and indirect effects of nitrogen addition and seasonal variation on (**a**) the overall protist community and (**b**) the protist functional groups (photoaut−trophs, parasites, phagotrophs) communities. Red arrows indicate negative effects; black arrows indicate positive effects.

Partial least squares path modeling (PLS-PM) demonstrated that N addition and season exerted direct and indirect effects on protist communities through soil nutrients and microbial diversity. The total community model exhibited acceptable predictive power (GoF = 0.58, SRMR = 0.067, R^2^ = 0.39), and the functional group model showed good fit (GoF = 0.618, SRMR = 0.073). Cross-validation results confirmed the stability and predictive reliability of both models, supporting the robustness of the inferred pathway structure. Total protist communities were primarily directly and positively influenced by microbial diversity, with a path coefficient of 0.23 for fungal diversity. Soil nutrients had no significant direct effect but exerted indirect positive effects by regulating microbial diversity. N addition had a direct negative effect on total protists (path coefficient = −0.18). Season operated indirectly through plants and soil nutrients, with plant diversity showing the strongest positive effect on total communities (path coefficient = 0.48). The model explained 39% of the variation in total protist communities (Figure 6a).

Functional groups exhibited distinctly different response patterns. N addition indirectly inhibited phototrophic protists by negatively affecting soil nutrient PC2 (path coefficient = −0.38), while season had a direct positive effect. Parasitic protists were indirectly regulated by N addition. Soil nutrient PC1 promoted fungal diversity, which in turn positively influenced the community (path coefficient = 0.46). Phagotrophic protists were most strongly directly affected by plant diversity (path coefficient = 0.53). The explanatory power of functional group models ranked as follows: parasites (R^2^ = 0.61) > phagotrophs (R^2^ = 0.38) > phototrophs (R^2^ = 0.34). Overall, N addition primarily exerted indirect effects through soil nutrients, whereas season operated mainly indirectly through plants (Figure 6b).

## 4. Discussion

### 4.1. Response of Soil Protist Communities to N Addition

Nitrogen (N) addition significantly altered soil protist community structure in the larch forest of the Greater Khingan Mountains, consistent with findings from agricultural ecosystems and our first hypothesis [30]. We also observed pronounced seasonal differences: Shannon diversity in CK was significantly higher than in all N addition treatments in July, whereas diversity indices in MN and HN treatments significantly exceeded those in CK and LN treatments in September. This divergence may be associated with greater soil acidification and NH_4_^+^ accumulation under higher N input during summer, conditions that are often linked to physiological stress in protist communities [31]; improved temperature and moisture conditions in autumn, combined with accumulated N promoting microbial proliferation, provided abundant food resources for protists, resulting in a lagged stimulation effect [32]. Previous studies have demonstrated that protist responses to N deposition vary significantly among different soil components [33]. The pronounced seasonal divergence observed in this study likely reflects the distinct adaptive strategies of protist communities under temporally heterogeneous microhabitat conditions. Seasonal variations in temperature, soil moisture, plant phenology, and microbial resource availability were associated with differences in protist community composition and functional-group patterns across sampling periods [8]. During summer, elevated temperatures and intensified microbial activity may have exacerbated soil acidification and ammonium stress under high N input, thereby imposing stronger constraints on protist growth and survival. In contrast, autumn litter accumulation and enhanced resource availability likely alleviated environmental stress and promoted the recovery of protist communities, particularly among phagotrophic and phototrophic functional groups.

Unlike findings from grassland ecosystems where low N significantly reduced protist diversity, our LN treatment showed no apparent negative effects and even exhibited stimulatory effects in certain seasons [13]. This discrepancy is primarily attributable to the inherently N-poor characteristics of cold-temperate forest soils. Low temperatures and slow organic matter decomposition in this region result in chronically low soil N availability, endowing the ecosystem with certain buffering and adaptive capacities to moderate N input [34]. Unexpectedly, TN concentrations in several N-addition treatments, particularly the LN treatment in May, were lower than those in the CK treatment. This phenomenon may reflect accelerated N turnover rather than simple N accumulation under long-term N input conditions. Chronic N addition may stimulate plant nutrient uptake, microbial N consumption, and N leaching losses, while simultaneously accelerating soil organic matter decomposition and nutrient mineralization [11]. These processes can reduce soil organic N storage despite increased inorganic N availability [14]. Consistent with this interpretation, NH_4_^+^-N concentrations increased under N addition treatments, whereas TN and TC contents generally declined, suggesting enhanced nutrient cycling and depletion of soil organic matter pools under long-term N enrichment. Previous research has identified N limitation as a key factor regulating organic matter decomposition and plant growth in cold-temperate forests, and global meta-analyses have confirmed that the negative effects of N addition on soil eukaryotic diversity vary by ecosystem type [35]. In strongly N-limited systems, moderate N input can enhance microbial diversity—our findings align with these conclusions [36].

### 4.2. Responses of Protist Functional Groups to N Addition

Different functional groups based on nutritional strategies exhibited significantly divergent responses to N addition, supporting our second hypothesis, though some response patterns differed from expectations. Phagotrophic and phototrophic protists were sensitive to N addition, whereas parasitic protists showed no significant response—differences that can be interpreted through the life history characteristics of each group.

As major consumers in the soil microbial food web, phagotrophic protists are often closely associated with the availability and diversity of bacterial and fungal communities [37,38]. Existing research confirms the tight coupling between bottom-up resource regulation and top-down predatory control within soil food webs [39]. In this study, fungal diversity was positively associated with phagotrophic protist diversity, further substantiating the importance of bottom-up regulatory mechanisms [40].

Phototrophic protists showed the most sensitive response, with highly significant differences in community structure (*p* = 0.002). This is closely related to their metabolic plasticity combining both autotrophic and heterotrophic capabilities [4]. Moreover, dominant groups such as Sphaeropleales are known to be sensitive to light and nutrient changes, and N addition may be associated with changes in understory vegetation cover, which could in turn be linked to variation in phototrophic protist communities [37]. Previous studies have demonstrated that soil phototrophic and consumer protists are driven by multiple environmental factors; our findings further support this view [39]. The highest phototrophic diversity observed in MN in September, together with increased abundance of Grossglockneriidae and Trinematidae, may be associated with autumn light changes and soil nutrient redistribution.

Parasitic protists showed no significant response to N addition, likely because their population dynamics are primarily regulated by host communities rather than by direct responses to soil N input [41]. The dominant parasitic taxa detected in this study were primarily affiliated with Pseudoperkinsidae, a lineage that includes species with parasitic or host-associated lifestyles. Their population dynamics may depend more strongly on host availability than on direct responses to soil N enrichment. These host organisms may exhibit relatively stable population dynamics under moderate N addition, thereby buffering parasitic protists against rapid environmental fluctuations [14]. Additionally, most parasitic taxa detected herein belong to transitional groups between free-living and facultative parasites, and can improve tolerance to environmental stress via life-history strategies including cyst formation [42,43]. Divergent responses across functional groups demonstrate that trophic strategies govern protist adaptation to long-term N enrichment. As key microbial predators, phagotrophic protists shape soil food webs by altering microbial grazing pressure and nutrient turnover alongside changes in their diversity and community composition. By comparison, phototrophic protists are more susceptible to fluctuations in nutrient supply and understory light, while parasitic protists are mainly limited by host dynamics.

### 4.3. Factors Associated with Soil Protist Community Changes Under N Addition

Based on Spearman correlation analysis and partial least squares path modeling (PLS-PM), protist community variation was associated with both abiotic soil properties and biotic community characteristics, which is generally consistent with our third hypothesis. However, the explanatory power of the models was moderate, indicating that additional unmeasured environmental and biological factors may also contribute to protist community variation. Notably, variations in Shannon diversity and taxon richness of soil protists are closely linked to the ecological traits of different functional groups rather than representing simple changes in community size across N addition and seasons. Phagotrophic protists, as key microbial predators, showed diversity shifts consistent with their role in regulating nutrient mineralization and responding to prey availability and food-web stability [8,39]. Phototrophic protists exhibited higher sensitivity to N enrichment and seasonal variation, likely reflecting their mixotrophic metabolism and strong dependence on light conditions, nutrient availability, and ammonium stress [4,37]. In contrast, parasitic protists maintained relatively stable diversity along the N gradient, consistent with their host-dependent life strategy that buffers them against environmental disturbance [41,43]. Overall, α-diversity metrics of total and functional protist groups can serve as effective indicators of microbial food-web dynamics and soil nutrient cycling in boreal forest ecosystems [10,15].

Regarding soil physicochemical properties, total protist diversity was significantly negatively correlated with SOC, DOC, and pH—a result that differs from findings in subalpine ecosystems [44]. This discrepancy likely stems from the unique environmental context of cold-temperate forests: high carbon content soils typically correspond to lower temperatures and slower organic matter turnover rates, creating oligotrophic conditions that constrain protist growth and metabolic activity [45]. Meanwhile, soil acidification induced by N addition in this study had not yet reached toxic thresholds; instead, it created niche space for acid-tolerant groups, conferring them competitive advantages and ultimately resulting in the observed negative correlations [31]. Examining differential effects of N forms, ammonium N showed significant inhibitory effects on phototrophic protists, consistent with findings on cellular toxicity of high ammonium concentrations [46]. The significant positive correlation between nitrate N and phagotrophic protists indicates that N addition can directly regulate protist communities by improving soil N availability and by modulating microbial food web structure [47].

Among biotic factors, fungal diversity showed relatively strong positive associations with protist communities and parasitic protists in the PLS-PM analysis. These patterns suggest that fungal communities may be linked to protist community variation within soil food webs, although the observational nature of the dataset does not allow causal relationships to be established [48,49]. Bacterial diversity was correlated only with phototrophic protists, possibly reflecting close ecological associations between mixotrophic flagellates and bacteria [12,50]. However, the explanatory power of the PLS-PM for bacterial diversity was relatively low (R^2^ = 0.05–0.07), indicating limited predictive capacity of the model for this variable. Therefore, the ecological significance of bacterial diversity in regulating protist communities should be interpreted cautiously. Plant diversity was positively associated with phagotrophic protists; previous studies have suggested that plant communities are often linked to microbial community composition through root exudates and litter inputs, which may further be associated with protist community variation through bottom-up trophic relationships [12,51,52]. The trend of increasing plant biomass under low and medium N but decreasing under high N was broadly consistent with changes observed in phagotrophic protists. This correspondence may reflect potential linkages among plant, microbial, and protist communities, although the relatively moderate model explanatory power indicates that these relationships should be interpreted cautiously. It should be noted that the PLS-PM models explained only a moderate proportion of community variation (R^2^ = 0.34–0.61). Therefore, the inferred pathways should be interpreted as statistical associations rather than definitive ecological mechanisms. Additional unmeasured environmental, biological, and temporal factors may also contribute to the observed variation in protist communities.

In summary, this study systematically elucidated the mechanisms by which N addition affects soil protist communities in cold-temperate larch forests, enriching research data on soil protists in cold-temperate forests and providing a scientific basis for predicting microbial food web responses in boreal forests under global N deposition. Nevertheless, several limitations of this study should be acknowledged. First, the present study was primarily based on amplicon sequencing data, which provided limited information regarding the functional activities and trophic interactions of protist taxa under N addition. Moreover, DNA-based 18S rRNA sequencing could not distinguish metabolically active cells from dormant cysts, resting stages, or extracellular DNA derived from dead cells; therefore, the apparent stability of certain protist functional groups, particularly parasitic protists, may partly reflect the persistence of non-active DNA signals rather than exclusively representing active community responses to N addition. Second, this study was conducted within a single cold-temperate forest ecosystem, and thus the generality of the observed patterns across different forest types and climatic regions remains uncertain. Third, the temporal scale of this study is limited, as it was based on a single-year sampling campaign despite the long-term establishment of the N addition experiment; consequently, interannual variability was not captured, and caution is required when extrapolating the results to longer temporal dynamics. Fourth, the litter layer was not removed during N addition and sampling, and interception by *Larix gmelinii* litter may have reduced the effective N input to the 0–10 cm mineral soil layer, potentially leading to deviations in soil N availability. In addition, soil temperature and litter input were not independently manipulated, which may have indirectly influenced protist community responses. Finally, although significant associations among soil properties, microbial communities, and protists were identified, direct causal relationships could not be fully resolved. Future studies incorporating metagenomics, functional assays, multi-ecosystem comparisons, litter-removal manipulations, and multi-year time series observations will help to better elucidate the ecological mechanisms underlying protist responses to long-term N deposition.

## 5. Conclusions

Within a long-term nitrogen (N) addition experimental platform in a *Larix gmelinii* forest, soil protist communities sampled during the 2023 growing season exhibited differences in community structure, diversity, and functional-group composition among N addition treatments, with responses varying across sampling periods. Correlation analysis and PLS-PM indicated that protist community variation was associated with soil physicochemical properties, plant biomass, and microbial community characteristics, particularly fungal diversity. However, these relationships should be interpreted as statistical associations rather than causal mechanisms. Because protist communities were sampled only during a single growing season, the relative contributions of long-term nitrogen addition and interannual climatic variability cannot be distinguished. Furthermore, as treatment effects were evaluated based on experimental N addition levels rather than measured soil N availability, the observed patterns should not be interpreted as quantitative dose–response relationships. Future multi-year monitoring combined with direct measurements of soil nitrogen availability will be necessary to assess the temporal stability of these patterns and better understand protist community responses to N enrichment.

## Figures and Tables

**Table 1:** Chi-square values from mixed-effects models analyzing nitrogen addition, seasonal variation, and their interaction on soil microorganisms (bacteria and fungi).

Response Variable	df	N Deposition		df	Season		df	N × Season	
		*F*	*p*		*F*	*p*		*F*	*p*
Bac-Shannon	3	4.89	0.006	2	14.02	<0.001	6	1.73	0.135
Bac-Richness	3	8.93	<0.001	2	2.87	0.072	6	2.51	0.038
Fun-Shannon	3	5.28	0.004	2	0.45	0.641	6	3.19	0.014
Fun-Richness	3	9.76	<0.001	2	3.98	0.029	6	1.58	0.174

Note: Shannon diversity was analyzed using linear mixed-effects models (LMMs), and richness was analyzed using generalized linear mixed-effects models (GLMMs) with a negative binomial distribution. Statistical significance of fixed effects was evaluated using Type II *F*-tests.

**Table 2:** Chi-square values of mixed-effects models of nitrogen addition, seasonal variation, and their interaction on soil protists and functional groups.

Response Variable	df	N Deposition		df	Season		df	N × Season	
		χ^2^	*p*		χ^2^	*p*		χ^2^	*p*
Shannon diversity	3	18.568	<0.001	2	0.149	0.928	6	8.869	0.175
Taxon richness	3	37.892	<0.001	2	2.508	0.285	6	23.557	<0.001
Phagotrophs Shannon	3	12.927	0.005	2	2.479	0.29	6	11.758	0.067
Phagotrophs richness	3	9.846	0.02	2	14.719	<0.001	6	25.428	<0.001
Parasites Shannon	3	3.408	0.331	2	0.397	0.819	6	0.349	0.998
Parasites richness	3	5.982	0.112	2	12.498	0.002	6	11.997	0.064
Phototrophs Shannon	3	8.763	0.033	2	4.821	0.09	6	7.615	0.267
Phototrophs richness	3	10.319	0.016	2	6.627	0.036	6	8.793	0.184

Note: Shannon diversity was analyzed using LMMs; taxon richness was analyzed using negative binomial GLMMs.

## Data Availability

The processed datasets supporting the conclusions of this study, including OTU abundance tables, taxonomic annotation files, metadata, and analysis-related files, are publicly available on Figshare at: https://doi.org/10.6084/m9.figshare.32049777.

## Supplementary Materials

The following supporting information can be downloaded at: https://www.mdpi.com/article/10.3390/ani16111734/s1, Tables S1 and S2: Main physical and chemical properties of soil under nitrogen addition. Table S3: The main physical and chemical properties of soil and microbial diversity under nitrogen addition. Figure S1: Correlation heatmap between soil physicochemical properties, plant taxa richness, microbial diversity and α-diversity of total protist community and different functional groups.
