# Supplementary material for: Effects of a 12-Year Nitrogen Addition Experiment on Protist Communities in a Boreal Forest, Heilongjiang Province, China
Source: Animals (Basel). 2026 Jun 4;16(11):1734. doi: 10.3390/ani16111734 (PMC13255705; doi:10.3390/ani16111734)
Supplement: Supplementary file 1 [file animals-16-01734-s001.zip › animals-4317114-supplementary.pdf]

Supplementary Material

Effects of a 12-Year Nitrogen Addition Experiment on Protist Communities in a Boreal Forest, Heilongjiang Province, China

Gang Fu[1], Guancheng Liu[1, 2], Ligong Wang[3], Yuguo Gao[4], Zhicheng Yao[5], Yajuan Xing[1, 2*], Qinggui Wang[1, 2*]

1 School of Life Science, Qufu Normal University, Qufu, 273165, China

2 Shandong Key Laboratory of Wetland Ecology and Biodiversity Conservation in the Lower Yellow River, Qufu Normal University, Qufu, 273165, China

3 Daxing'anling Institute of Agriculture and Forestry, Jiagedaqi, 165000, China

4 Shandong Weishan Lake Wetland Ecosystem National Positioning Observation and Research Station, Jining, 272000, China

5 Zibo Forestry Protection and Development Center, Zibo, 255000, China

* Correspondence: yajuanxing@163.com (Y.X.); qgwang1970@163.com (Q.W.)

Table S1 Main physical and chemical properties of soil under nitrogen addition

treatment		SWC（%）	pH	TN（$g·kg^{-1}$）	TC（$g·kg^{-1}$）	Plant
May	CK	45.22±6.46a	4.61±0.07b	1.87±0.39a	47.60±2.09a	95.31±4.58c
	LN	30.74±2.71b	4.86±0.05a	0.96±0.06b	24.66±1.82d	107.98±4.23a
	MN	32.78±2.51b	4.84±0.12a	1.18±0.09b	30.56±4.14b	101.90±4.23b
	HN	31.16±2.06b	4.41±0.21c	1.20±0.13b	26.95±0.68c	82.11±3.33d
Jul	CK	23.81±1.47a	5.01±0.04a	2.27±0.30a	45.81±10.38a	101.53±4.38a
	LN	16.41±1.98c	5.07±0.02a	1.22±0.11d	31.00±4.11c	98.70±4.11a
	MN	20.27±1.11b	5.12±0.10a	1.68±0.14b	38.38±1.47b	98.97±4.05a
	HN	19.99±1.20b	4.79±0.09b	1.42±0.13c	32.99±2.80c	85.34±3.41b
Sep	CK	29.38±2.42a	4.92±0.01a	2.03±0.26a	42.81±9.12a	95.60±4.18b
	LN	20.09±0.82c	4.89±0.10a	1.26±0.09c	28.87±3.33b	101.59±4.21b
	MN	24.34±1.14b	4.83±0.09b	1.46±0.12b	30.67±4.62b	104.51±4.23a
	HN	23.62±1.89b	4.56±0.15c	1.32±0.14c	27.12±1.09b	92.43±3.88c

Note: Soil **pH** value was measured by mixing soil with water in a ratio of 1:2.5, allowing it to stand for 30 minutes, and then taking the upper layer of clear liquid for pH measurement using a pH meter (Sartorius PB-10, Germany). Soil moisture content (**SWC**) was determined by drying method. Fresh soil was dried in a 60°C oven until constant weight was achieved, and weight difference was calculated. Total carbon (**TC**) of soil was determined by placing 0.1 g of air-dried soil in a porcelain combustion boat, and then conducting a dry combustion at 1100°C using a carbon-nitrogen elemental analyzer (Multi C/N 3000, Analytik Jena, Germany). Dissolved organic carbon (**DOC**) of the soil was measured by shaking for 6 hours at a shaking speed of 3000 $r·min^{-1}$, followed by centrifugation at 3000 $r·min^{-1}$ and filtration through a 0.45 μm filter membrane, and then determined using a carbon-nitrogen elemental analyzer in a vertical furnace. Microbial biomass carbon (**MBC**) of soil was determined by chloroform fumigation - K_2SO_4 extraction method. Take 2 portions of 5 g of fresh soil, one portion was dark fumigated at 25°C for 24 hours, other portion was not fumigated. Add 0.5 $mol·L^{-1}$ K_2SO_4 to the soil in a water-to-soil ratio of 1 : 4 for extraction, and then perform a 300 $r·min^{-1}$ shaking filtration and determine using a carbon-nitrogen elemental analyzer. Total nitrogen (**TN**) of soil was determined by adding 1 g of soil and 1 mL of deionized water, 5 mL of H_2SO_4, and boiling until transparent, then diluting to a certain volume, and determining using a continuous flow analyzer (Skalar Analytical B.V., Netherlands) according to the Kjeldahl nitrogen determination method. Ammonium nitrogen (**NH_4^+-N**) and nitrate nitrogen (**NO_3^--N**) were extracted with 2 $mol·L^{-1}$ KCl within 24 hours after sampling, and filtered. Filtrate was determined using the above continuous flow analyzer according to molybdenum-sulfur antimony colorimetric method. Total phosphorus (**TP**) was determined by dissolving the soil in H_2SO_4-H_2O_2, filtering the solution, and determining using the above continuous flow analyzer according to the molybdenum-sulfur antimony colorimetric method. Different lowercase letters (a, b, c, d) indicate significant differences among treatments within the same month (Tukey's HSD test, $p < 0.05$). Values are means ± standard deviation.

Table S2 Main physical and chemical properties of soil under nitrogen addition

treatment		TP ($g\cdot kg^{-1}$)	MBC ($mg\cdot kg^{-1}$)	DOC($g\cdot kg^{-1}$)	NO_3^--N($mg\cdot kg^{-1}$)	NH_4^+-N($mg\cdot kg^{-1}$)
May	CK	0.57±0.11 a	972±144 a	0.20±0.03 b	1.48±0.21 b	1.03±0.10 d
	LN	0.52±0.04 a	729±187 ab	0.19±0.01 b	1.29±0.18 b	1.41±0.36 c
	MN	0.43±0.04 b	660±73 b	0.19±0.01 b	1.61±0.16 ab	4.02±1.04 b
	HN	0.42±0.05 b	476±14 c	0.23±0.02 a	1.62±0.35 a	7.37±1.84 a
Jul	CK	0.78±0.06 a	395±117 a	0.35±0.02 b	2.92±0.75 b	1.94±0.28 c
	LN	0.49±0.03 b	393±45 a	0.37±0.02 a	3.71±0.46 a	1.65±0.46 c
	MN	0.58±0.04 a	434±74 a	0.33±0.03 b	4.20±0.58 a	13.11±3.69 a
	HN	0.45±0.04 b	369±75 a	0.36±0.05 a	3.34±0.52 ab	10.29±2.27 b
Sep	CK	0.63±0.05 a	477±127 a	0.35±0.02 b	1.48±0.21 b	1.03±0.10 c
	LN	0.40±0.02 b	266±96 b	0.37±0.02 a	1.29±0.18 b	1.41±0.36 c
	MN	0.41±0.03 b	423±94 a	0.33±0.03 b	1.61±0.16 ab	4.02±1.04 b
	HN	0.38±0.03 b	225±10 b	0.36±0.05 a	1.62±0.35 a	7.37±1.84 a

Note: Different lowercase letters (a, b, c, d) indicate significant differences among treatments within the same month (Tukey's HSD test, $p < 0.05$). Values are means ± standard deviation.

Table S3 The main physical and chemical properties of soil and microbial diversity under nitrogen addition

treatment		SOC ($g{\cdot}kg^{-1}$)	Bac-Shannon	Bac-Richness	Fun-Shannon	Fun-Richness
May	CK	27.08±3.15c	6.35±0.17b	1431.33±159.80bc	3.31±0.35bc	184.00±27.75cd
	LN	30.74±3.54a	6.45±0.07ab	1530.00±50.84ab	3.32±0.31bc	225.33±30.02bc
	MN	28.76±7.04ab	6.37±0.12b	1367.67±121.06cd	3.39±0.18bc	227.00±22.00bc
	HN	27.53±2.29bc	6.17±0.26c	1219.00±180.58d	3.60±0.29ab	211.00±41.79cd
Jul	CK	27.41±2.51c	6.59±0.06a	1802.67±281.05a	2.95±0.42c	200.00±19.14cd
	LN	34.34±1.92a	6.59±0.10a	1540.33±67.05ab	3.62±0.08ab	270.00±7.02ab
	MN	34.05±3.72a	6.77±0.06a	1730.67±77.44a	4.13±0.21a	295.67±51.06a
	HN	28.52±4.28b	6.53±0.11a	1452.67±110.36bc	3.57±0.32ab	220.67±21.98bc
Sep	CK	29.53±0.50a	6.56±0.05a	1527.67±27.43ab	3.30±0.24bc	237.67±19.17bc
	LN	27.23±2.04b	6.66±0.03a	1671.67±24.25ab	3.77±0.19ab	289.33±29.87a
	MN	27.38±1.15b	6.78±0.01a	1797.00±42.10a	3.26±0.47bc	262.67±20.41ab
	HN	23.66±2.48c	6.36±0.30b	1453.33±222.11bc	3.48±0.15abc	206.33±27.83cd

Note: Different lowercase letters (a, b, c, d) indicate significant differences among treatments within the same month (Tukey's HSD test, $p < 0.05$). Values are means ± standard deviation.

Figure S1 Correlation heatmap between soil physicochemical properties, plant taxa richness, microbial diversity and *α*-diversity of total protist community and different functional groups

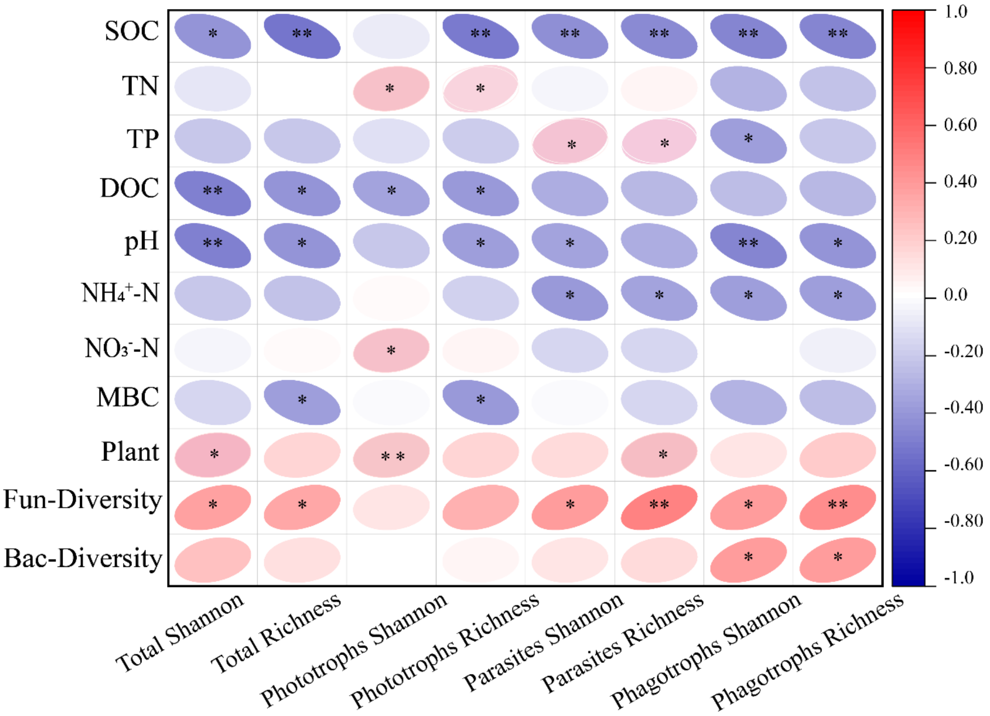

Note: Red indicates positive correlation, and blue indicates negative correlation. Deeper color and larger ellipse area represent higher absolute value of correlation coefficient. * $p < 0.05$, significant correlation; ** $p < 0.01$, extremely significant correlation. SOC: soil organic carbon; TN: total nitrogen; TP: total phosphorus; DOC: dissolved organic carbon; NH_4^+-N: ammonium nitrogen; NO_3^--N: nitrate nitrogen; MBC: microbial biomass carbon; Plant: plant taxa richness.
